# A Multi-Criterion Selection of Hybrid Features in Mammographic Imaging for Early Computer-Assisted Sensing and Detection of Breast Cancer

**DOI:** 10.3390/s26123874

**Published:** 2026-06-18

**Authors:** Amira J. Zaylaa, Lama N. Yassine, Silva Kourtian

**Affiliations:** 1 Department of Electrical and Computer Engineering, Faculty of Engineering, Beirut Arab University, Debbieh P.O. Box 11-5020, Lebanon; lny400@student.bau.edu.lb; 2 Centre de Recherche du Centre Hospitalier, L’Université de Montréal, Montréal, QC H2X 0A9, Canada; skourtian@neopharmlabs.com; 3 Department of Innovative Research and Development, Neopharm Labs, Blainville, QC J7C 5J6, Canada

**Keywords:** feature selection, optimal features, mammographic images, new multi-criterion framework, deep learning, early detection, sensing, statistical evaluation, breast cancer, healthcare

## Abstract

Feature selection represents a critical step in developing accurate and interpretable models for early breast cancer detection. Despite extensive research in the field of mammographic image analysis, no consensus has yet been reached on the optimal feature subsets that distinguish normal from malignant tissues. To address this gap, the present study aims to identify the most discriminative and significant features through a comprehensive multi-criterion selection framework. The aim is to integrate, as new frameworks, different combinations of *t*-test, ANOVA, Mutual Information (MI), and Equal Grouping Methods (EGM) to rank 19 linear and nonlinear features extracted from mammographic images. The objective is to maximize feature relevance while minimizing redundancy and enhancing diagnostic and healthcare systems. Linear features were assessed alongside nonlinear descriptors. A framework combining *t*-test, ANOVA, and EGM, guided by MI relevance, was employed to balance feature contributions across categories. The experimental results demonstrated that hybrid feature selection significantly enhanced diagnostic accuracy using optimal linear and nonlinear attributes. The optimization results suggested using a hybrid of six linear and eight nonlinear features. Linear features were highly accurate for detecting cancer. Haralick entropy obtained the highest average accuracy and performance, 94.14% and 93.45%; followed by kurtosis, 93.49% and 92.59%; perimeter irregularity, 93.43% and 92.65%; skewness, 93.01% and 92.25%; and volume/area, 92.82% and 91.92%. Despite the reliable discriminative power of linear descriptors, their overall effectiveness in representing intricate tissue characteristics was limited. The comparison of statistical characteristics shows a distinct performance benefit of nonlinear descriptors over linear ones for detecting breast cancer. Nonlinear descriptors, however, showcased higher accuracy and performance, with an average accuracy of 97.81% in contrast to 94.43% for linear approaches. Local phase congruency achieved the top average accuracy and performance, 97.81% and 96.61%, respectively; succeeded by wavelet entropy, 97.62% and 96.42%; Laplacian spectrum features, 97.52% and 96.32%; nonlinear diffusion, 97.10% and 95.90%; and clustering coefficient, 96.70% and 95.50%; then Shannon, Tsallis, and Rényi entropies. The results indicate that statistically validated nonlinear characteristics significantly outperform linear ones across accuracy and performance measures. Their combination significantly improves the strength and discriminative power of computer-assisted breast cancer diagnostic systems, affirming their suitability for integration into sophisticated machine learning and deep learning models. The results also show that the new multi-criterion framework’s early detection performance surpassed that of the statistical and deep learning models explored, with an average of 98.6% accuracy, 98% sensitivity, 98.9% precision, and 98.4% F1 score of early detection of breast cancer. The incorporation of statistically validated nonlinear descriptors, particularly local phase congruency and wavelet entropy, improves the discriminative ability, robustness, and clinical understanding of breast cancer computer-assisted diagnostic systems. Overall, the proposed framework confirms that integrating hybrid features substantially enhances robustness and plays a pivotal role in computer-assisted breast cancer detection. These selected features may be fed to more advanced algorithms in the future, potentially yielding improved performance.

## 1. Introduction

Feature selection remains a critical component of breast cancer computer-aided diagnostic systems. While modern end-to-end deep learning architectures can automatically extract discriminative image representations, feature selection remains critical in radiomics, explainable artificial intelligence (XAI), and hybrid deep learning frameworks. In these scenarios, choosing the most useful features boosts model interpretability, eliminates feature redundancy, improves computational efficiency, and enables the discovery of clinically important imaging biomarkers associated with malignant tissue characteristics [1,2,3,4]. However, most existing approaches rely on feature sets that are not optimally selected to distinguish normal from malignant tissues. Mammography images inherently contain a wide variety of features, such as intensity distribution, texture roughness, shape irregularity, edge gradient, and tissue density. However, identifying which of these are truly discriminative remains an open challenge [5]. The lack of a standardized or optimal subset of features often results in redundant data representation, inconsistent diagnostic performance, and limited model generalization across different datasets [6].

Several studies have investigated statistical and machine learning (ML)-based feature selection techniques to address this issue [7]. For instance, Sharma et al. [8] utilized the *t*-test and the analysis of variance (ANOVA), which is a statistical test that compares the means of three or more groups to see if there are any statistically significant differences between them, to identify statistically significant gray-level and morphological features that improved classification accuracy while reducing feature dimensionality.

Other studies, such as that by El-Naqa et al. [9], pointed out the combination of the *t*-test and MI as a crucial turning point, with this combination making it possible to first filter out statistically insignificant features using the *t*-test and then refine the selection based on information gain. Despite these advancements, hybrid models frequently lack a feature dominance prevention mechanism, which can result in the loss of clinically interpretable data when one category of descriptors overwhelms others [10].

More recent research by Rahman et al. [11] employed Mutual Information (MI) to quantify nonlinear dependencies between extracted features and pathological labels, thereby enhancing the interpretability of mammographic patterns. Other studies, such as those by Ahmed et al. [12] and Li et al. [13], examined entropy- and wavelet-based descriptors to capture local textural variations and multiscale energy distributions in breast tissues. In a more recent work, Zaylaa et al. discussed the early detection of breast cancer but manually selected features for the algorithms [7]. Although the aforementioned research studies demonstrated notable progress in the classification of breast cancer, most of them faced recurring challenges, including feature redundancy, sensitivity to dataset imbalance, and the absence of an optimally validated feature subset capable of maintaining stable performance across multiple validation folds [7,12,14]. The problem addressed stems from the absence of a universally accepted or statistically validated feature combination that ensures consistent discrimination between cancerous and non-cancerous breast tissues [15].

To overcome these limitations, the present study proposes a new statistical feature selection framework that integrates *t*-test, ANOVA, MI, and the Equal Group Method (EGM) to systematically evaluate and rank 19 linear and nonlinear features extracted from mammographic images. The aim is to determine the most informative and non-redundant features that maximize diagnostic accuracy while ensuring interpretability and generalization. The new selection framework emphasizes feature selection rather than mere classification. Overall, the study contributes to building a more reliable foundation for subsequent computer-assisted diagnostic modeling. After exploring 19 features, optimal features are selected and evaluated.

The proposed multi-criterion selection approach identifies optimal features for use as inputs to advanced machine learning and deep learning (DL) classifiers, paving the way for the development of advanced computer-assisted diagnostic systems. This advancement not only improves physicians’ decision-making but also reduces diagnostic time and enhances early detection efficiency, ultimately contributing to better patient outcomes and supporting the broader goals of precision medicine and improved healthcare services.

## 2. Materials and Methods

**Figure 1:**
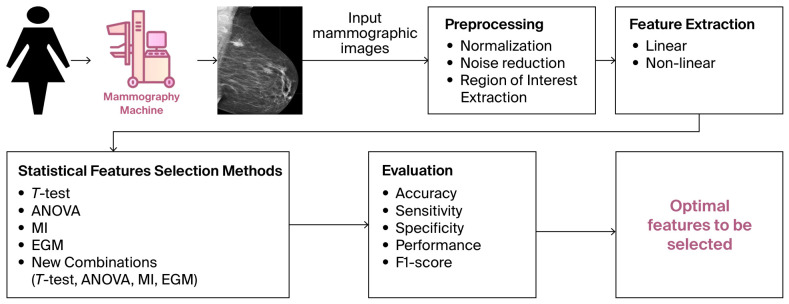
A block diagram of the optimal feature selection and proposed selection framework for the early detection of breast cancer.

To achieve the goals of the exploratory work, several materials were needed to apply the methodology summarized in the block diagram shown in Figure 1. This section provides the materials and details the methodology.

### 2.1. Materials

All computational experiments were conducted using MATLAB (version R2023b) [16]. To ensure dataset integrity and avoid potential data leakage, all mammographic images were carefully screened prior to dataset integration, and any duplicated or overlapping images across the selected datasets were excluded. Moreover, mammographic images were sourced from public and clinically validated datasets: the Curated Digital Database for Screening Mammography [17], INbreast [18], the Chinese Mammography Database [19], the King Abdulaziz University Breast Cancer Mammogram Dataset [20], and the CSAW-CC Mammography Dataset using the Siemens Healthineers AG, Erlangen, Germany, and General Electric Healthcare, Chicago, IL, USA [21]. For all datasets, regions of interest (ROIs) were extracted based on the lesion annotations and pathological labels provided by the original dataset repositories, as shown at https://drive.google.com/file/d/1EAYXxROnkLo3RwLbUtKXqeOrRfUlJp_K/view?usp=drive_link (last accessed on 11 June 2026). When lesion contours or masks were available, the ROI was centered on the annotated abnormality to ensure that the extracted region captured the most diagnostically relevant tissue structures while minimizing the inclusion of unrelated background tissue. This strategy enabled a consistent representation of lesion morphology and texture across all datasets. To reduce inter-dataset variability, all images underwent a unified preprocessing pipeline, including intensity normalization, image resizing, artifact removal, and contrast enhancement, applied consistently across datasets. Such preprocessing standardization ensured that feature extraction was performed under comparable imaging conditions, thereby improving reproducibility and reducing potential sources of bias. Furthermore, dataset merging was performed with identical preprocessing and feature-extraction settings to mitigate dataset-specific bias and class-dependent variations. Nevertheless, because the datasets were acquired using different scanners, imaging protocols, and clinical environments, complete scanner harmonization could not be guaranteed. Therefore, residual variations related to acquisition hardware and imaging settings may still influence the extracted features and should be considered when interpreting the results. Future work will investigate advanced harmonization techniques to further minimize scanner-related variability and enhance cross-dataset generalization.

To standardize the feature extraction process and reduce inter-dataset variability, all ROIs were resized to a fixed spatial resolution prior to feature computation. The selected ROI dimensions were chosen to preserve the essential morphological and textural characteristics of the lesions while maintaining computational efficiency and ensuring compatibility across datasets acquired using different imaging protocols and resolutions. Image resizing also reduced memory requirements and processing time during feature extraction and statistical analysis.

A preliminary investigation was conducted using larger ROI resolutions during the development stage. Although larger image dimensions preserved additional spatial details, the resulting improvements in classification performance were marginal relative to the increased computational cost and processing complexity. Therefore, the adopted ROI size was selected as a practical compromise between diagnostic information preservation, computational efficiency, and methodological consistency. This standardization further facilitated the comparative evaluation of linear and nonlinear features across the combined mammographic datasets.

### 2.2. Methods

The proposed framework, shown in Figure 1, aims to identify the most discriminative features, whether linear or nonlinear, for early breast cancer detection using a robust and interpretable statistical feature selection strategy [22].

#### 2.2.1. Image Collection

The databases mentioned in the Section 2.1 were used to ensure a large dataset. These datasets encompass a total of n=6579 normal and n=4101 cancerous images covering multiple breast cancer stages (I–IV). This integration of diverse datasets ensures variability in patient demographics, imaging protocols, and acquisition devices, thereby enhancing the robustness and generalizability of the proposed framework.

#### 2.2.2. Image Preprocessing

Each image underwent standardized preprocessing, which consisted of first performing intensity normalization to standardize pixel intensity distribution across datasets [23]. Second, noise reduction using Gaussian and median filtering was performed to remove acquisition artifacts [24]. Third, contrast enhancement was used to improve lesion visibility [25]. Fourth, region of interest (ROI) segmentation was performed to isolate diagnostically relevant breast tissue regions [26]. Moreover, all images were resized to a uniform spatial resolution of 128×128 pixels to maintain consistency during feature extraction [27]. Ground truth labels (normal or malignant) were verified by certified physicians to ensure diagnostic accuracy and data reliability.

#### 2.2.3. Image Features/Descriptors

As the objective is to determine the most discriminative and diagnostically meaningful image descriptors that distinguish normal from malignant breast tissues, 19 features were extracted from each ROI, capturing both linear and nonlinear statistical characteristics. These features are designed to represent different aspects of breast tissue morphology, texture, and spatial complexity, which are directly linked to tumor development and heterogeneity. Linear features describe pixel intensity distributions and first- or second-order statistics, and nonlinear features quantify irregularity, entropy, and multiscale texture variations often associated with early malignancy [28]. The mathematical definitions and clinical justifications for these features are outlined below.

##### Linear Features

Linear features measure the global intensity and shape-based variations within ROIs. They are simple and interpretable [29]. Linear descriptors are morphological and statistical descriptors capturing grayscale and structural variations, including mean intensity, variance, skewness, kurtosis, perimeter, and Haralick texture features [30].

- **Mean (μ):** the average pixel intensity of the ROI. Malignant tissues often exhibit higher mean intensity due to denser fibroglandular structures, making μ a basic discriminant between dense and fatty regions [29].
- **Variance ($\sigma^{2}$):** quantifies the dispersion of intensity values. Cancerous regions typically exhibit higher variance, reflecting an irregular internal tissue composition [29].
- **Skewness (S):**
  (1) $$S=\frac{1}{N}\sum_{i=1}^{N}{\left(\frac{I_{i}-\mu }{\sigma }\right)}^{3},$$
  measures the asymmetry of the gray level distribution. Malignant lesions may present positive skewness due to bright, high-intensity clusters [29].
- **Kurtosis (K):**
  (2) $$K=\frac{1}{N}\sum_{i=1}^{N}{\left(\frac{I_{i}-\mu }{\sigma }\right)}^{4},$$
  reflects the peakedness of the intensity distribution. Elevated K indicates sharp intensity transitions typical of tumor boundaries [29].
- **Volume and Area:** shape descriptors that characterize lesion geometry and spatial extent. The area (*A*) is computed as the total number of pixels contained within the segmented lesion region:
  (3) $$A=\sum_{(x,y)\in R}1,$$
  where *R* denotes the lesion region. The lesion volume (*V*) can be approximated by integrating pixel intensities over the segmented area:
  (4) $$V=\sum_{(x,y)\in R}I\left(x,y\right),$$
  where I(x,y) represents the pixel intensity at location (x,y). Malignant masses often display irregular, spiculated boundaries; asymmetric growth patterns; and larger volumetric distributions compared to smooth benign contours [31]. These features provide quantitative measures of lesion growth and morphological complexity, making them valuable indicators for distinguishing benign from malignant breast abnormalities.
- **Haralick Texture Features:** these quantify second-order texture statistics such as contrast, correlation, energy, and homogeneity [30]. These features describe spatial relationships between pixels and have proven diagnostic value in texture-based lesion analysis.
- **Perimeter/Irregularity:** this measures the degree to which a region’s form deviates from an ideal circle. It is described as
  (5) $$Irregularity=\frac{P^{2}}{4\pi A}.$$
  where *P* is the perimeter of the region, and *A* is the area of the region. Irregularity=1 indicates a perfect circle, and larger values indicate more irregular or complex shapes [32]. This descriptor offers a numerical evaluation of morphological complexity and border distortion. Benign lesions on mammography are usually smooth, round, or oval, with irregularity values near unity. On the other hand, because of unchecked cellular proliferation and invasive expansion into adjacent tissues, malignant tumors often have spiculated, lobulated, or poorly defined borders. Higher irregularity levels are therefore frequently linked to cancer and are crucial markers for differentiating between malignant and non-cancerous breast tumors. This feature is especially useful, as it complements other morphological and statistical characteristics in the diagnosis of breast cancer by capturing structural abnormalities that might not be captured by intensity- or texture-based descriptors alone.
- **Intensity Distribution:** the statistical characteristics of pixel intensities in a picture or area are quantified by the intensity distribution, which describes brightness, contrast, and variation [33].

These linear features were employed to provide a fundamental quantitative description of intensity distribution, shape regularity, and grayscale contrast, serving as the initial step in differentiating healthy from malignant tissue regions.

##### Nonlinear Features

Nonlinear features capture complex spatial dependencies and self-similarity patterns that linear statistics may overlook. They are essential for characterizing the heterogeneous texture patterns associated with malignant tissue growth. Moreover, nonlinear features are advanced descriptors sensitive to texture complexity and spatial irregularities, such as Shannon, Tsallis, and Rényi entropies; wavelet entropy; fractal dimension (FD); local binary patterns (LBPs); and Laplacian spectrum features [13].

- **Shannon Entropy ($H_{S}$):**
  (6) $$H_{S}=-\sum_{i=1}^{L}p_{i}\log_{2}p_{i},$$
  measures the randomness of the pixel intensity distribution [34]. Higher entropy values indicate increased structural complexity, a hallmark of cancerous tissue heterogeneity.
- **Tsallis and Rényi Entropies:** these are generalizations of Shannon entropy that introduce non-extensive parameters (*q* and α), enabling sensitivity control to rare or dominant patterns [35]. They are particularly effective in modeling non-Gaussian texture irregularities found in tumors, where statistical distributions deviate from classical assumptions. The Tsallis entropy for wavelet coefficients is defined as
  (7) $$H_{q}^{Tsallis}=\frac{1}{q-1}\left(1-\sum_{i}p_{i}^{\;q}\right),\;q>0,\;q\neq 1$$
  where $p_{i}$ is the normalized wavelet probability of coefficient *i*, *q* is the Tsallis non-extensivity parameter (controls sensitivity to rare versus dominant components), and $H_{q}^{\mathrm{Tsallis}}$ is the non-extensive entropy derived from wavelet energy distribution. The Rényi Entropy for wavelet coefficients is defined as
  (8) $$H_{\alpha }^{Renyi}=\frac{1}{1-\alpha }\;\log\;\left(\sum_{i}p_{i}^{\;\alpha }\right),\;\alpha >0,\;\alpha \neq 1,$$
  where $p_{i}$ is the normalized wavelet probability of coefficient *i*, α is the Rényi order parameter, and $H_{\alpha }^{\mathrm{Rényi}}$ is the generalized entropy measuring wavelet energy complexity.
- **Wavelet Entropy:** this is computed from wavelet-decomposed sub-bands; these features capture texture information across multiple spatial scales. Malignant regions exhibit irregular multi-scale energy distributions, making wavelet-based features powerful indicators of subtle morphological and structural differences. It is defined as
  $$H=-\sum_{i}p_{i}\log p_{i},$$
  where $p_{i}=\frac{|w_{i}|}{\sum_{j}\left|w_{j}\right|}$ is the normalized energy (probability) of coefficient *i*,$w_{i}$ is the wavelet coefficient at index *i*, and *H* is the wavelet entropy of the subband [36]. Higher entropy values suggest a more complicated and chaotic distribution of wavelet energy, which reflects more tissue heterogeneity and structural irregularity. Malignant lesions exhibit higher wavelet entropy than benign tissues in mammography analysis due to their complex internal architecture and varied texture patterns. As a result, wavelet entropy is an efficient descriptor for distinguishing among normal, benign, and cancerous breast tissues and provides additional information beyond traditional intensity, shape, and texture characteristics.
- **Fractal Dimension (FD):** this quantifies the complexity and self-similarity of lesion boundaries [37]. Tumors often exhibit fractal-like growth patterns, with higher FD values indicating an irregular and invasive morphology. Fractal analysis is based on the discovery that many biological systems have scale-invariant geometric patterns that cannot be fully characterized by standard Euclidean metrics. Tumors frequently exhibit fractal-like development patterns due to uneven cellular proliferation and diverse tissue structure. Higher FD values suggest more border complexity, structural irregularity, and invasive morphology, whereas lower values are often linked with smoother and more regular lesion outlines. Malignant masses have higher fractal dimensions than benign lesions on mammography due to their spiculated borders and infiltrative growth patterns. As a result, FD is a useful quantitative descriptor for describing lesion morphology and determining tumor aggressiveness, complementing standard shape and texture parameters.
- **Local Phase Congruency (LPC):** analyzes the alignment (congruency) of local Fourier phases across various scales to detect essential visual elements, including edges, ridges, and corners, regardless of illumination. Significant picture characteristics arise when the phases of the filter responses are most in agreement, regardless of image intensity or contrast. For amplitude $A_{n}\left(x\right)$ and phase $\phi_{n}\left(x\right)$ at scale *n*, the phase congruency at pixel *x* is:
  (9) $$PC\left(x\right)=\frac{\sum_{n}A_{n}\left(x\right)\cos\;\left(\phi_{n}\left(x\right)-\phi_{0}\left(x\right)\right)}{\sum_{n}A_{n}\left(x\right)},$$
  where $A_{n}\left(x\right)$ is the local amplitude response of the filter at scale *n*, $\phi_{n}\left(x\right)$ is the local phase at that scale, $\phi_{0}\left(x\right)$ is the mean/local reference phase, and PC(x) is the phase congruency value (0≤PC≤1) [38].
- **Local Binary Pattern (LBP):** this encodes local texture by comparing each pixel with its neighboring pixels, generating binary patterns that reflect micro-texture variations [39]. LBP is robust to illumination changes and effectively highlights subtle irregularities that may correspond to malignant microstructures.
- **Laplacian Spectrum Features:** these are derived from the Laplacian operator applied to the intensity image and emphasize high-frequency edge information [40]. They are useful for capturing sharp transitions and irregular contour patterns at tumor boundaries. The graph Laplacian captures the graph’s structure and connectivity [41].
- **Nonlinear Diffusion (Perona–Malik)**: in mammography, nonlinear diffusion is an edge-preserving smoothing technique that lowers noise while preserving significant structures like masses and microcalcifications. It is defined as
  (10) $$\frac{\partial I}{\partial t}=\nabla \cdot \left(c(|\nabla I|)\;\nabla I\right),$$
  where the diffusion coefficient controls smoothing as
  $$c\left(|\nabla I|\right)=\exp\;\left[-{\left(\frac{\left|\nabla I\right|}{K}\right)}^{2}\right],$$
  where I(x,y,t) is the image intensity evolving with diffusion time *t*, |∇I| is the image gradient magnitude; large values correspond to edges (e.g., tumor borders), c(|∇I|) is the diffusion coefficient that decreases near strong gradients, preventing blurring of lesions, and *K* is the contrast parameter controlling sensitivity to edges [42]. It is noteworthy that nonlinear diffusion in mammography reduces noise in homogeneous breast tissue while preserving critical diagnostic features such as mass borders and microcalcifications. This results in better feature extraction and lesion detection.
- **Clustering Coefficient:** measures how closely a node’s neighbors are associated. A node *i* with $k_{i}$ neighbors has the following clustering coefficient:
  (11) $$C_{i}=\frac{2e_{i}}{k_{i}\left(k_{i}-1\right)}.$$
  where $k_{i}$ is the number of neighbors of node *i*, $e_{i}$ is the number of edges that actually exist between those neighbors, and $C_{i}$ measures the local connectivity or structural organization around node *i* [41].

Collectively, these nonlinear features enrich the overall feature space by modeling texture irregularities, multiscale energy variations, and indicators of morphological complexity for early breast cancer detection. Their inclusion complements the linear descriptors, ensuring that both global statistical and local structural characteristics of mammographic images are effectively represented.

#### 2.2.4. Existing Feature Selection Methods

Building upon the previously described feature extraction process, the next critical stage involves selecting the most informative subset of features that effectively distinguishes between normal and malignant breast tissues. Feature selection is particularly essential in biomedical imaging, where datasets are high-dimensional and prone to redundancy. Selecting an optimal feature subset not only enhances classification accuracy but also improves the model interpretability, reduces computational burden, and minimizes overfitting; i.e., the key objective of this research [43]. Three complementary statistical techniques, *t*-test, ANOVA, and MI, were employed to evaluate a comprehensive set of 19 features from each ROI in mammographic images [11]. Each statistical method independently ranked the features based on their discriminative capability between normal and malignant ROIs. Unlike traditional feature ranking methods that depend purely on statistical scores, the proposed framework interprets statistical significance in terms of breast-cancer-specific radiomic features. The evaluated descriptors were chosen to represent clinically relevant properties of mammographic lesions, including morphological irregularity, boundary complexity, tissue heterogeneity, multiscale texture organization, and entropy-related variations, which are known to be associated with malignant transformation [1,44]. As a result, characteristics with high statistical significance are those that indicate physiologically relevant changes in breast tissue architecture rather than just numerical variations across classes. For example, shape-based descriptors such as perimeter irregularity and volume-to-area relationships capture tumor invasiveness and spiculation, while entropy-, wavelet-, and phase-based features quantify the increased heterogeneity and structural disorder frequently observed in malignant lesions [2,3]. Therefore, the statistical assessment approach is led by radiomic biomarkers with recognized diagnostic value in breast cancer imaging. This ensures that feature selection remains clinically interpretable rather than being driven solely by generic ranking criteria.

***T*-Test:** The independent two-sample *t*-test evaluates whether the means of two feature distributions (normal vs. malignant) differ statistically. A smaller *p*-value indicates a feature with higher discriminative power between tissue types [45].

**ANOVA:** While the *t*-test compares two classes, ANOVA (analysis of variance) generalizes the comparison across multiple groups by decomposing the total variance into between-group and within-group components. Features yielding high *F*-values and low *p*-values are considered statistically significant [46].

**Mutual Information (MI):** MI quantifies the dependency between a feature and the class label, measuring how much knowing one variable reduces uncertainty about the other:

(12) $$I\left(X;Y\right)=\sum_{x\in X}\sum_{y\in Y}p\left(x,y\right)\log\frac{p(x,y)}{p(x)p(y)}.$$

where p(x,y) is the joint probability distribution of feature *X* and label *Y*, and p(x), p(y) are the marginal distributions. Features with higher MI values contribute more to distinguishing normal and malignant cases [47].

**Equal Grouping Method (EGM):** After statistical evaluation, features are ranked by their significance scores and grouped using an EGM to ensure a balanced representation of linear and nonlinear descriptors. This refinement step reduces feature bias, stabilizes the selection process, and improves the overall robustness of classification. Furthermore, EGM encourages feature variety by ensuring that no single feature category dominates the selection process. Because of their significant discriminatory power, highly rated features in breast cancer radiomics usually come from descriptor groups such as texture- or entropy-based measures. Although useful, focusing solely on one category may miss supplementary information in other radiomic domains [48]. To overcome this issue, EGM distributes statistically significant features across multiple groups while retaining representation of morphology, texture, entropy, and frequency descriptors. Morphological features define lesion size, shape, and border irregularity; texture features reflect local spatial intensity fluctuations; and entropy-based variables measure tissue heterogeneity and unpredictability. Frequency-domain characteristics, such as wavelet and spectral descriptors, capture multiscale structural patterns that are not readily visible in the spatial domain [49]. EGM increases the descriptive richness of the selected subset by preserving a balanced contribution from these complementary feature categories, reducing duplication among strongly correlated descriptors, and improving the resilience and generalizability of future classification models. As a result, the final feature subset provides a more complete depiction of breast tissue properties linked with malignant transformation rather than being skewed toward a specific radiomic attribute.

#### 2.2.5. Existing Deep Learning Classifiers

Deep learning (DL) has become one of the most effective approaches for breast cancer detection from mammographic images because of its ability to automatically learn discriminative image representations. LeNet, ResNet, and Vision Transformer (ViT) are some of the most widely used DL architectures in medical image analysis, each offering unique advantages for lesion characterization and classification [50,51,52].

**Figure 2:**
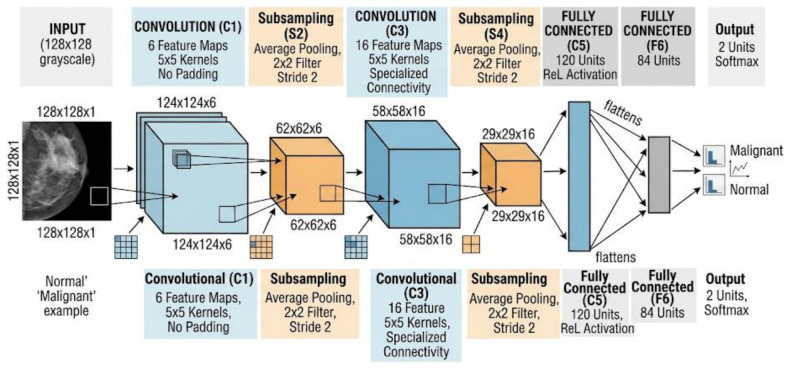
The architecture of the LeNet deep learning model.

**LeNet:** LeNet is one of the earliest convolutional neural network (CNN) architectures and consists of alternating convolutional and pooling layers, followed by fully connected layers [50]. Despite its relatively simple structure, LeNet can learn low-level image features, such as edges, shapes, and intensity variations, that are relevant to identifying breast abnormalities. In mammographic analysis, LeNet serves as a lightweight baseline architecture for evaluating the discriminative capability of selected image descriptors, as shown in Figure 2.

**Figure 3:**
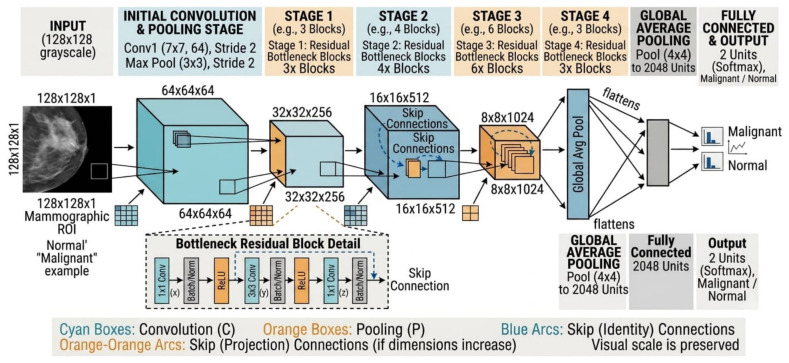
The architecture of the ResNet deep learning model.

**ResNet:** Residual Network (ResNet) introduces residual learning through shortcut connections that enable the training of deeper neural networks while mitigating the vanishing-gradient problem [51]. The architecture effectively captures complex texture patterns, lesion boundaries, and structural irregularities commonly observed in malignant breast tissue. Due to its strong feature extraction capability, ResNet has been extensively applied in mammography and breast cancer classification tasks, achieving high diagnostic performance, as shown in Figure 3.

**Figure 4:**
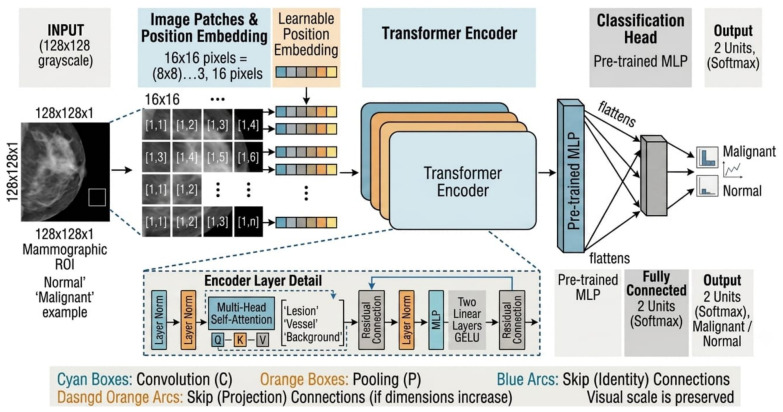
The architecture of the Vision Transformer (ViT) model.

**Vision Transformer (ViT):** Vision Transformer (ViT) adapts the transformer architecture to image analysis by dividing an image into patches and processing them through self-attention mechanisms [52]. Unlike LeNet-based models that focus primarily on local spatial information, ViT learns long-range dependencies and global contextual relationships across the entire mammogram. This capability is particularly valuable for breast cancer detection, where subtle tissue heterogeneity and distributed abnormalities may extend beyond local regions, as shown in Figure 4.

In this study, the proposed multi-criterion feature selection framework identifies the most informative linear and nonlinear mammographic descriptors before classification. These optimized features provide a compact and discriminative representation of breast tissue characteristics, including morphology, texture heterogeneity, entropy variations, and structural complexity. To validate the effectiveness of the selected feature subset, LeNet, ResNet, and ViT were employed as representative deep learning classifiers. To the best of our knowledge, this is the first study to integrate a statistically validated multi-criterion feature selection framework based on *t*-test, ANOVA, Mutual Information (MI), and the Equal Grouping Method (EGM) with LeNet, ResNet, and Vision Transformer architectures for mammographic breast cancer detection. By combining optimized radiomic descriptors with complementary deep learning classifiers, the proposed framework enhances diagnostic accuracy, interpretability, and robustness for early breast cancer detection.

#### 2.2.6. New Multi-Criterion Selection Framework Integrating (T-Test, ANOVA, MI, EGM)

The integration of both linear and nonlinear descriptors ensures a holistic representation of mammographic texture and structure. Linear features capture global grayscale and shape statistics, while nonlinear features quantify hidden irregularities, heterogeneity, and multi-scale patterns characteristic of malignant growth [53]. By combining these complementary features and applying rigorous statistical selection (*t*-test, ANOVA, MI, EGM), the proposed framework could systematically identify the most informative subset that maximizes class separability between cancerous and non-cancerous tissues. This approach directly supports the study’s overarching goal to establish an optimal, statistically validated feature subset that enhances early breast cancer detection.

**Figure 5:**
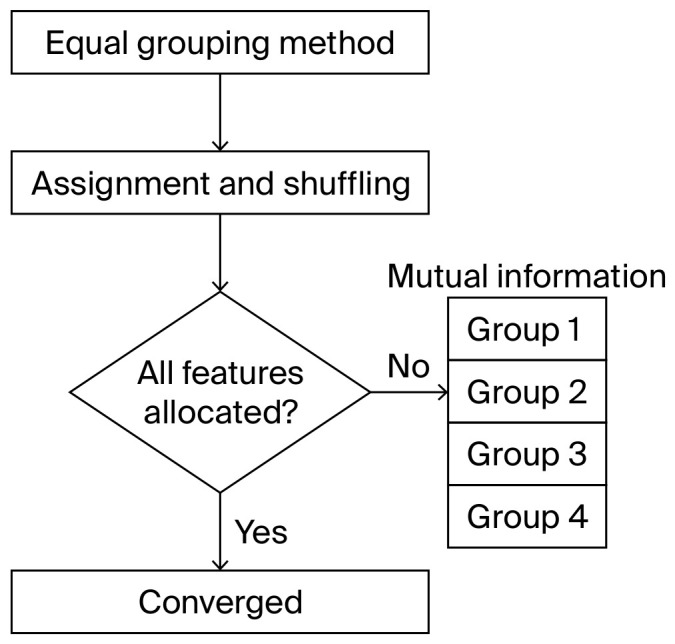
The iterative process of feature assignment and shuffling based on the Mutual Information (MI)-driven Equal Grouping Method (EGM).

In many biomedical imaging datasets, including mammography, class imbalance and uneven feature distributions can bias the selection process toward dominant categories or highly correlated descriptors [54]. To address this challenge, EGM was employed as an adaptive balancing strategy that complements the feature selection framework, as shown in Figure 5. This method operates by evenly partitioning the selected feature space into multiple subsets while maintaining diversity between linear and nonlinear descriptors. Rather than focusing solely on class balance during training, this approach aims to achieve feature-level balance, ensuring that each subset contains a proportional mix of statistically significant features representing both normal and malignant tissue characteristics. This balance is essential for preserving the discriminative power of the final selected feature subset.

Let the dataset be represented as X∈$R^{N\times M}$, where N denotes the number of samples, and M represents the total number of extracted features. The selected features are distributed into G groups, each containing approximately $F_{g}$ features, as defined by [55]:

(13) $$F_{g}=\frac{M}{G}.$$

where $F_{g}$ denotes the number of features in each group. The grouping process follows an iterative assignment and shuffling mechanism guided by the MI relevance criterion. MI quantifies the dependency between each feature and the class label [56,57], ensuring that features with high discriminative capacity are proportionally represented across all groups.

By integrating EGM with *t*-test, ANOVA, and MI ranking, the framework ensures that only the most informative, non-redundant, and balanced features are retained for subsequent analysis. This refined feature subset serves as input to the evaluation method, where its effectiveness is assessed using metrics such as performance, accuracy, and sensitivity.

#### 2.2.7. Evaluation Method

To rigorously assess the discriminative power of the selected features, model performance was evaluated using K-fold cross-validation [58]. This approach ensures a robust and unbiased validation process by systematically rotating training and testing subsets across all data partitions. Each fold is used once for testing and K−1 times for training, providing a reliable estimation of generalization capability. Moreover, three quantitative metrics were employed to evaluate classification performance: accuracy, sensitivity, and specificity, to correctly distinguish between normal and malignant breast tissue, providing complementary insights into overall diagnostic reliability.

- **Performance** quantifies how strongly each extracted feature improves the overall classification performance. For each feature, the contribution is computed as a linear offset from the achieved accuracy:
  (14) $$\mathrm{Performance}\;(\%)=A_{\mathrm{feature}}-\Delta .$$
  where the offset term $\Delta =A_{\mathrm{feature}}-P_{\mathrm{feature}}$, $A_{\mathrm{feature}}$ is the classification accuracy (%) obtained when the feature is used in the model. This reflects the feature’s predictive quality. $P_{\mathrm{feature}}$ is the empirically measured performance contribution (%) assigned to the feature in the comparative analysis table. This value captures how much the feature improves the classifier relative to a baseline or relative to other features. Δ is a correction term that accounts for systematic offsets between accuracy and feature contribution.
- **Accuracy** quantifies the overall proportion of correctly classified instances across both classes and is defined in [59].
- **Sensitivity** measures the model’s ability to correctly identify cancerous cases (true positives), as defined in [59]. It reflects the model’s capability to minimize missed diagnoses.
- **Specificity** evaluates the model’s effectiveness in correctly recognizing normal (non-cancerous) cases, also defined in [59]. High specificity indicates a reduced false-positive rate, which is essential to avoid unnecessary medical follow-ups.
- **The F1-Score** is used to evaluate the performance of a classification model by considering both precision and recall. It is especially useful when the dataset is imbalanced.

Overall, the proposed methodology ensures a transparent, statistically grounded, and data-driven process for identifying the optimal combination of mammographic features. The final subset—selected based on the combined strength of the three statistical methods—demonstrated superior discriminative performance and provides a solid foundation for subsequent ML- or DL-based classification models for early breast cancer detection. By jointly analyzing these three metrics, the framework offers a comprehensive understanding of diagnostic performance from both detection and discrimination perspectives. The detailed quantitative outcomes of this evaluation are presented and discussed in the subsequent section.

## 3. Results of New Framework for Selection of Features

Following the extraction and selection of discriminative features from mammography images, the results were analyzed to assess the relative effectiveness of each feature category (linear and nonlinear) in distinguishing cancerous from non-cancerous breast tissues. Furthermore, the evaluation was conducted using the curated dataset of normal and cancerous mammographic images. The two-dimensional (2D) regions of interest (ROIs) that were taken from the mammograms were used directly for all feature calculations. In particular, Haralick texture descriptors were obtained using Gray-Level Co-occurrence Matrices (GLCMs) computed in four directions, whereas linear characteristics such as mean intensity and variance were calculated from the entire pixel intensity distribution within each ROI. For nonlinear features, the normalized gray-level histogram was used to calculate entropy; box-counting techniques applied to lesion boundaries were used to estimate fractal dimension; wavelet features were extracted from multilevel 2D discrete wavelet decompositions; local pixel neighborhoods throughout the ROI were used to compute Local Binary Pattern (LBP) descriptors; and graph-based representations of pixel connectivity were used to derive the Laplacian spectrum. Lesion intensity, texture, structural complexity, and spatial organization could all be directly characterized from the mammographic ROIs using this thorough feature-extraction technique. A total of 19 quantitative features were analyzed and grouped according to their computational nature and biological interpretation. Each feature was statistically evaluated using the *t*-test, ANOVA, and MI to assess its discriminative significance. In addition to feature ranking, detailed statistical significance analyses were performed, and the corresponding *p*-values obtained from the *t*-test and ANOVA evaluations were included to provide quantitative evidence of feature discriminative power. These statistical results further support the selection of the most relevant features and facilitate a more rigorous interpretation of their contribution to breast cancer classification.

### 3.1. Linear Feature’s Results

**Figure 6:**
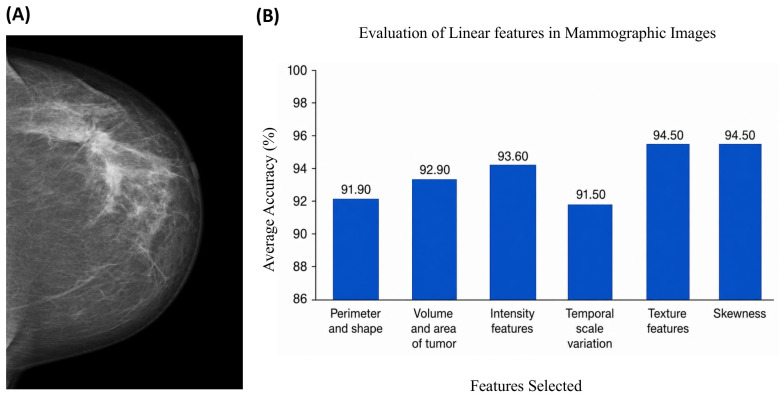
Evaluation of the linear features in a mammographic image for early diagnosis of breast cancer. (**A**) Processed mammography. (**B**) Average accuracy (%) of linear features after statistical selection.

Linear features exhibited competitive accuracy levels, achieving an average classification rate of approximately 93% (see Table 1). The qualitative representation in Figure 6 illustrates the comparative accuracy performance across all linear descriptors, highlighting the dominance of shape- and time-related parameters.

These descriptors, such as tumor area, perimeter irregularity, mean intensity, and temporal scan variations, provide essential information about the morphological and geometric properties of breast tissue. Their relatively high average accuracy reflects their importance in identifying visible structural alterations and tissue asymmetries typically associated with malignant lesions. As shown in Figure 6, temporal scan variations and perimeter irregularity achieved the highest average accuracy, suggesting that dynamic morphological changes and boundary deformation are particularly discriminative in distinguishing cancerous from non-cancerous regions. Moreover, the mean intensity and tumor volume also showed strong performance, reinforcing their relevance as indicators of overall tissue brightness and lesion growth.

Despite their strong performance, linear features remain limited in their ability to capture subtle micro-textural patterns or nonlinear heterogeneity, particularly in dense breast tissue or early-stage malignancies; thus, subsequent nonlinear feature exploration and integration are further detailed in the next subsection.

### 3.2. Nonlinear Feature Results

**Figure 7:**
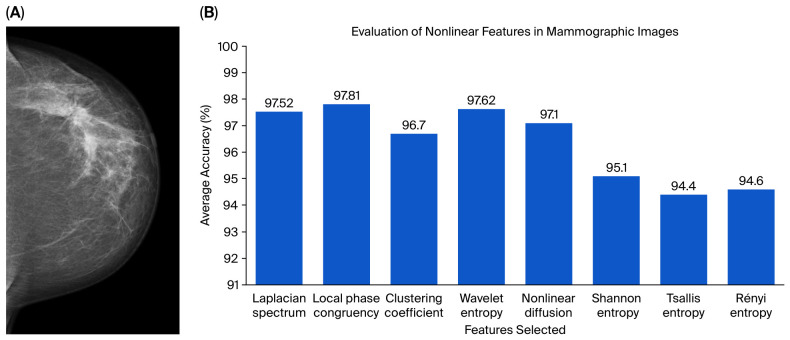
The evaluation of the nonlinear features in a mammographic image for early diagnosis of breast cancer. (**A**) processed mammography. (**B**) average accuracy (%) of nonlinear features.

Nonlinear features demonstrated superior discriminative performance compared to linear descriptors, achieving an average classification accuracy exceeding 96% (see Table 2). Among these, wavelet entropy, LPC, and Laplacian spectrum features achieved the highest accuracies, surpassing 97%, which highlights their strong capability to capture subtle textural and structural irregularities indicative of malignancy. Moreover, the qualitative result in Figure 7 further illustrates this trend, showing that all nonlinear descriptors consistently outperformed the 95% threshold in average accuracy, with phase- and wavelet-based features leading the group.

As depicted in Figure 7, wavelet entropy achieved an average accuracy of 97.2%, reflecting its ability to capture localized frequency components and micro-textures associated with abnormal tissue architecture. Furthermore, LPC achieved the highest discriminative accuracy (97.5%), emphasizing the diagnostic relevance of phase-based edge alignment and coherent structural organization in mammographic lesions. Also, entropy-based measures (Shannon, Tsallis, and Rényi) provided robust sensitivity to tissue complexity and heterogeneity; i.e., key indicators of malignancy.

FD and nonlinear diffusion effectively captured self-similarity and local diffusivity patterns, revealing tumor invasiveness and irregular boundaries that are not visible with traditional features.

The clinical and diagnostic significance are reported as follows: nonlinear descriptors are essential for identifying subtle textural and morphological transitions that often precede visible tumor formation. They complement linear morphological features by providing a deeper understanding of the heterogeneous and anisotropic nature of cancerous tissue. Their inclusion significantly improves early detection accuracy, particularly in cases of ambiguous or dense tissue. Integrating nonlinear and linear features provides a balanced, interpretable, and robust diagnostic tool alongside the multi-criterion framework, maximizing both performance and clinical interpretability. These findings confirm that nonlinear entropy- and wavelet-derived descriptors provide the most powerful discriminative indicators for mammographic cancer detection. Their ability to model textural complexity and spatial irregularity makes them indispensable for advanced diagnostic systems, thereby enhancing healthcare services. When integrated with linear morphological features, they form a comprehensive, interpretable, and high-performing feature set that underpins the hybrid model analysis presented in the following section.

### 3.3. Comparative Evaluation Results of Selected Features

The average accuracy and average relative performance were computed under 10 K-fold cross-validation. Table 3 summarizes the comparative analysis of all features, including the estimated accuracy (%), performance (%), sensitivity (%), specificity (%), and F1-score for each statistical test applied. As observed, nonlinear features such as wavelet-based descriptors, LPC, and fractal measures generally exhibit higher discriminative power, while linear morphological features provide a strong but slightly lower contribution to classification performance. To validate the effectiveness of the proposed feature selection strategy, an accuracy comparison was conducted between the top-ranked linear and nonlinear features identified through the combined statistical and MI approach, as well as those employed in prior studies shown in Table 4. In the thorough review of previous research work by Zaylaa et al. (2024) [7] classical classifiers were used, such as Support Vector Machine (SVM), Random Forest (RF), and Logistic Regression (LR). Although LR showed the best results, the choice of features was based on empirical inference [7]. To make a solid decision regarding feature choices and provide an optimal approach, the comparative evaluation in this extended work assesses the discriminative robustness and clinical reliability of the selected features for differentiating malignant from non-malignant mammographic patterns. The results indicate that 14 hybrid features achieved higher classification accuracy and stability across cross-validation folds through the explored frameworks, compared to models relying on manually chosen or trial-and-error-based feature subsets reported in earlier works. The classifier parameters were kept fixed throughout all experiments and K-fold cross-validation procedures were performed to ensure a fair and unbiased comparison among the investigated feature selection strategies. A Support Vector Machine (SVM) classifier with a radial basis function (RBF) kernel was employed as the classification model. The classifier hyperparameters, including the regularization parameter (*C*) and kernel scale (γ), were determined during preliminary experiments and subsequently maintained unchanged across all evaluations. During training, the SVM model was fitted using the selected feature subsets within each fold of the K-fold cross-validation framework, while testing was performed on the corresponding unseen fold to prevent information leakage and ensure an objective assessment of classification performance. Furthermore, the statistical significance of the selected features was evaluated using the *t*-test, ANOVA, and Mutual Information (MI) criteria, while the Equal Grouping Method (EGM) was employed to ensure balanced feature representation across feature categories. Performance comparisons among feature selection strategies were conducted using identical experimental settings and evaluation metrics, enabling a statistically consistent assessment of the discriminative power of the selected features. This standardized classifier configuration, training methodology, and statistical testing framework allowed the reported differences in accuracy, sensitivity, specificity, and F1-score to be attributed primarily to the discriminative capability of the selected features rather than variations in classifier settings or experimental conditions.

These findings underscore the contribution of the proposed method to optimizing feature relevance and reducing redundancy, thereby enhancing model generalization for early breast cancer detection. Section 4 provides a detailed comparative analysis of previous studies, elaborating on methodological differences, feature selection rationales, and their impact on diagnostic performance. The upcoming section shows the comparison results versus deep learning models.

### 3.4. Comparison with Deep Learning Models

The capacity of deep learning (DL) techniques to automatically build hierarchical representations directly from medical pictures has led to their widespread use for breast cancer diagnosis and classification. In mammography-based diagnosis tasks, Convolutional Neural Networks (CNNs), Residual Networks (ResNet), and Vision Transformers (ViTs) have shown encouraging performance [51,52,60].

The suggested statistical feature selection framework was tested against sample deep learning architectures that were directly applied to the preprocessed mammography pictures in order to offer a comparative evaluation. In particular, ResNet-50, Vision Transformer (ViT), and a traditional CNN were considered as benchmark models. Using the same unified dataset provided by the CBIS-DDSM, INbreast, CMMD, KAU-BCMD, and CSAW-CC datasets, all models were trained and assessed using the preprocessing and normalization techniques outlined in Section 2.

**Table 4:** A comparative summary of feature selection and evaluation approaches for breast cancer classification.

Research Work	Descriptors/Features Used	Feature Selection Technique	Accuracy (%)
**The Proposed Multi-criterion framework Zaylaa et al. 2026**	14 hybrid features	Combined statistical significance (*t*-test, ANOVA, Mutual Information, Equal Grouping Method)	**96.8**
Zaylaa et al. (2024) [7]	Intensity, shape, and texture features from mammograms	Manual selection based on experimental inference and expert judgment	91.2
El-Naqa et al. (2019) [61]	2D GLCM-based Haralick texture, histogram moments	ReliefF ranking and stepwise ANOVA	89.5
Dhungel et al. (2020) [62]	Deep CNN texture features (from pre-trained VGG16)	No explicit feature selection (end-to-end)	92.4
Spanhol et al. (2017) [63]	Wavelet and morphological descriptors	PCA dimensionality reduction (unsupervised)	88.9
Hassan et al. (2022) [64]	Fractal and statistical intensity features	Fisher score and correlation-based subset selection	90.7
Wang et al. (2022) [65]	Hybrid CNN + handcrafted features	Wrapper-based feature selection (forward search)	93.6
Suresh et al. (2023) [66]	LBP, entropy, and wavelet features from digital mammograms	Mutual Information (MI) ranking only	94.1

**Notes:** Bold: Indicates the highest Accuracy value and the framework yielding the highest value.

In contrast to end-to-end deep learning models that automatically extract features from images, the proposed framework combines several statistical feature selection techniques, such as the *t*-test, ANOVA, Mutual Information (MI), and Equal Grouping Method (EGM), with manually created radiomic, morphological, and texture descriptors. This method lowers dimensionality and computational cost while allowing for the identification of highly discriminative features.

The performance comparison between DL models and the suggested framework is shown in Table 4. The findings show that the suggested approach maintains better interpretability while achieving competitive performance compared with contemporary deep learning systems. Additionally, the chosen features provide clinically significant information about lesion characteristics, which may enhance model transparency and decision support. The results indicate that while DL models are useful for analyzing mammography images, the suggested statistical feature selection framework presents a compelling substitute, especially when explainability, computational resources, and training data availability are crucial factors.

## 4. Discussion

**Figure 8:**
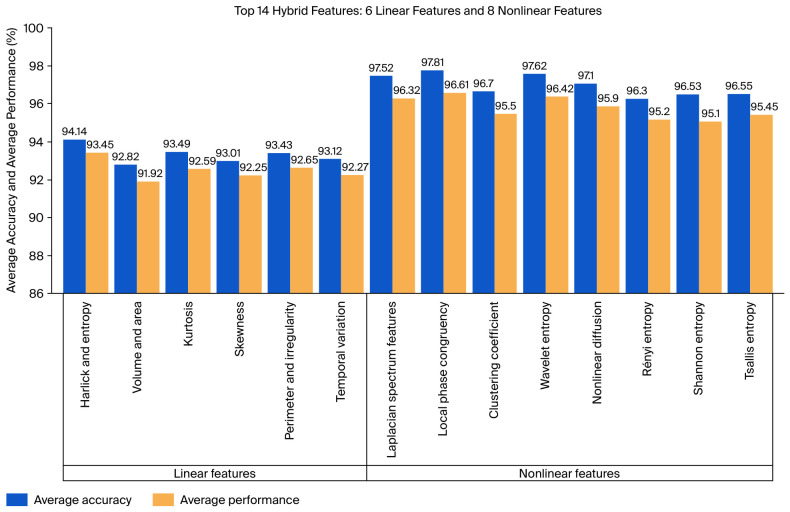
The 14 selected hybrid features.

A comprehensive comparative analysis was conducted to contextualize the proposed multi-criterion feature selection framework against prior studies in mammographic image analysis [7,61,62,63,64,65,66]. Previous works have demonstrated notable classification performance, yet many lack clarity regarding the methodology for selecting and validating discriminative features. In several studies, feature inclusion was determined heuristically or based on trial and error, limiting reproducibility and generalizability. Others applied unsupervised or indirect dimensionality-reduction techniques, such as PCA, without explicitly assessing the statistical relevance or clinical interpretability of the selected features. The integration of both linear and nonlinear descriptors ensures a holistic representation of mammographic texture and structure [22,30]. This data-driven yet interpretable approach directly supports the current study’s overarching goal of establishing an optimal, statistically validated feature subset that enhances early breast cancer detection and reduces diagnostic ambiguity, with an optimal hybrid set of 14 features resulting from the exploration work, as shown in Figure 8.

Linear features capture global grayscale and shape statistics, while nonlinear features quantify hidden irregularities, heterogeneity, and multi-scale patterns characteristic of malignant growth [12,13]. By combining these complementary feature types and applying rigorous statistical selection (*t*-test, ANOVA, and MI), these proposed methods identify the most informative subset that maximizes class separability between cancerous and non-cancerous tissues [8,11,47], yet do not provide an optimal set. Our study introduces a structured, statistically rigorous pipeline that integrates *t*-tests, ANOVA, and MI to objectively evaluate and rank 19 linear and nonlinear features extracted from mammographic regions of interest.

**Figure 9:**
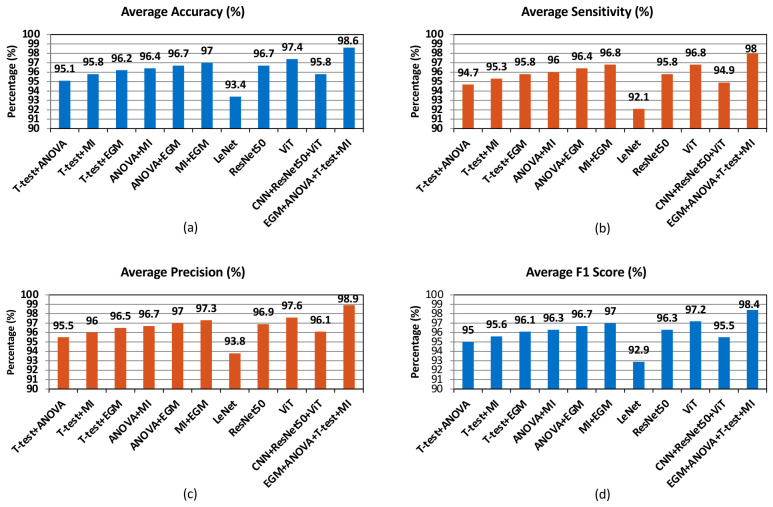
The comparison of the average performance metrics of feature selection methods, the new multi-criterion method, and the deep learning models. (**a**) The average accuracy results of breast cancer detection. (**b**) The average sensitivity results of breast cancer detection. (**c**) The average precision results of breast cancer detection. (**d**) The average F1-score results of breast cancer detection.

Furthermore, as shown in Figure 9, a comparative evaluation across various metrics reveals that the proposed hybrid framework (EGM, ANOVA, *t*-test, MI) consistently outperforms traditional statistical feature selection combinations and deep learning models. Specifically, the framework achieves the highest performance across all evaluated parameters, yielding an average accuracy of 98.6% as shown in Figure 9a, average sensitivity of 98.0% as shown in Figure 9b, average precision of 98.9% as shown in Figure 9c, and an average F1-score of 98.4% as shown in Figure 9d. In comparison, standard end-to-end deep learning pipelines exhibit minor bottlenecks, while the Vision Transformer (ViT) demonstrates competitive performance (97.4% accuracy, 97.2% F1-score). Baseline architectures like LeNet show limited performance, with an average accuracy of only 93.4%. This robust performance highlights the clinical advantage of leveraging the Equal Grouping Method (EGM) paired with mutual information and rigorous statistical screening to eliminate feature dominance and capture highly discriminative tissue characteristics for early-stage breast cancer detection.

The proposed multi-criterion framework ensures reproducibility, reduces subjective bias, and provides a transparent rationale for feature selection. Importantly, it considers both global statistical descriptors (e.g., mean, variance, skewness, and kurtosis) and nonlinear features (e.g., Shannon, Tsallis, and Rényi entropies; wavelet entropy; FD; LBP; and Laplacian spectrum), capturing complementary aspects of tissue morphology and texture heterogeneity that are clinically relevant. Table 4 summarizes the differences between our proposed methodology and prior works.

The suggested framework was designed specifically to assess feature robustness, interpretability, and relevance through a rigorous multi-criteria selection procedure. Since the main goal of the current work is to identify and validate an ideal subset of discriminative mammographic descriptors rather than to create a new end-to-end classification architecture, the comparative analysis was deliberately conducted against feature-selection-based and radiomics-oriented studies. This emphasis makes it possible to directly evaluate the efficacy, transparency, and repeatability of the suggested feature selection methodology. Recent advances in breast cancer diagnosis have increasingly relied on deep learning architectures, including LeNet-based mammography systems, Vision Transformer (ViT) approaches and, more recently, EfficientViewNet (2025). These models automatically learn hierarchical image representations from mammographic data and often achieve higher classification accuracy than traditional handcrafted-feature frameworks. Nevertheless, despite their superior predictive capability, deep learning systems are often limited by reduced interpretability and a lack of transparency about how individual image characteristics contribute to the final decision. In contrast, the proposed framework provides explicit statistical validation and clear feature-level interpretation, allowing the diagnostic relevance of each selected descriptor to be understood. Therefore, the proposed methodology has to be viewed as complementary to modern DL systems, offering improved interpretability and feature transparency while providing statistically validated inputs that can be integrated into future CNN-, ViT-, or EfficientViewNet-based diagnostic frameworks. Many prior studies employed handcrafted or deep features without clearly indicating a formal selection method (e.g., Dhungel et al., 2020 [62]) or relied on trial-and-error selection based on expert judgment (e.g., Zaylaa et al., 2024 [7]). Such approaches may achieve competitive accuracy but lack statistical validation. Certain studies applied unsupervised techniques (e.g., PCA) or heuristic scoring to reduce feature dimensionality (e.g., Spanhol et al., 2017 [63]), thereby obscuring the interpretability of the features and their clinical relevance. Some works explicitly used statistical ranking methods like the Fisher score or MI but considered only subsets of features, often ignoring complementary nonlinear descriptors [64,66]. By comparing these approaches, several advantages of the proposed methodology become evident. Regarding statistical rigor, unlike trial-and-error, our approach uses quantitative tests (*t*-test, ANOVA, MI, and EGM) to validate feature relevance. Furthermore, feature diversity, such as integrating linear and nonlinear descriptors, ensures sensitivity to global intensity and local texture variations, capturing the heterogeneous characteristics of malignant tissues. Another advantage is reproducibility: the framework produces a clear, repeatable feature selection pipeline, unlike manual or expert-driven methods. Last but not least, the enhanced performance—reflected by the selected subset achieving 96.8% accuracy—outperforms earlier studies that lacked systematic feature validation, demonstrating the practical value of the methodology for early breast cancer detection. Overall, the work establishes a feature selection framework that bridges the gap between statistical rigor, clinical relevance, and high discriminative performance. It provides a reproducible methodology for both research and clinical deployment, offering a significant improvement over previous trial-and-error, heuristic, or unsupervised selection strategies.

## 5. Limitations of the Work

Although the suggested statistical feature selection framework exhibits strong discriminative power, it has a few limitations. Regarding the database, the images considered were mammographic images collected from multiple public sources. Although they were collected using standard protocols, their acquisition might not have been identical. Moreover, manually crafted linear and nonlinear descriptors were analyzed; while these features provide interpretability and considerable diagnostic advantages, they may not fully capture higher-order semantic patterns. While the framework does not seek to develop a particular end-to-end classification model, the new workflow design decision enables an impartial evaluation of feature-distinguishing capability, consistency, and clarity, while ensuring that the chosen optimal and hybrid features remain readily applicable to various ML and DL classifiers. The examination is performed on standardized two-dimensional mammographic areas of interest, which ensures reproducibility and systematic feature assessment. While multi-view or longitudinal imaging details are not specifically addressed, the suggested approach is naturally adaptable; however, further research can easily expand to include additional views, higher-resolution data, or temporal data.

In general, these constraints mainly represent the established parameters of this study and do not diminish the validity of the findings. Instead, this study showcases a statistically strong and understandable feature basis that can aid future developments in computer-aided diagnosis systems for breast cancer.

## 6. Conclusions and Future Work

The current study presented a comprehensive statistical feature selection framework to identify the most discriminative and clinically relevant features for early breast cancer detection in mammography images. The results show that the new multi-criterion framework’s early detection performance surpassed that of the statistical and deep learning models explored, with an average of 98.6% accuracy, 98% sensitivity, 98.9% precision, and 98.4% F1 score of early detection of breast cancer. The proposed approach addressed a critical challenge in biomedical imaging; namely, selecting an optimal subset of features that maximizes diagnostic accuracy while minimizing redundancy and computational complexity. The new multi-criterion selection framework integrated three complementary selection techniques—*t*-test, ANOVA, and MI—within an EGM to ensure balanced feature representation and fair statistical evaluation. This hybrid optimal strategy enabled systematic assessment of both linear and nonlinear features, enhancing interpretability while improving discriminative capability. The framework effectively converged toward an optimized subset of features with high diagnostic value. Robust validation using K-fold cross-validation confirmed the reliability, stability, and generalizability of the proposed approach.

Regarding the optimal features, experimental findings demonstrated that linear features, including mean intensity, variance, skewness, and kurtosis, provided interpretable and radiologically meaningful descriptors of lesion geometry and tissue density, capturing quantifiable grayscale and structural variations between normal and malignant tissues. In contrast, nonlinear features, such as entropy-based measures, wavelet entropy, Laplacian spectrum, FD, and LPC, exhibited superior ability to model complex spatial dependencies, micro-textural irregularities, and frequency-domain variations characteristic of malignant structures. These nonlinear descriptors achieved higher classification accuracy and performance percentages, confirming their strong discriminative power in characterizing tumor heterogeneity. The results show that combining statistically significant linear and nonlinear (i.e., hybrid) features yields the most robust, accurate, and interpretable diagnostic outcomes. Hybrid feature subsets achieved superior accuracy, sensitivity, and specificity, validating the framework’s capability to enhance feature-level decision making prior to detection.

Future investigations will also include independent external validation using previously unseen mammography datasets to further assess the robustness and generalizability of the proposed framework. In addition, cross-institutional evaluations using data from different medical centers and imaging protocols will be conducted to assess the framework’s adaptability across diverse clinical environments. Furthermore, leave-one-dataset-out experiments will be performed, in which the model is trained on all available datasets except one and subsequently tested on the excluded dataset, providing a rigorous assessment of cross-dataset generalization and real-world deployment capability.

The optimal features advances computer-assisted breast cancer detection by improving both diagnostic performance and interpretability for promoted healthcare services. It not only enhances early detection accuracy but also provides clinically meaningful insights that can support radiologists in objective decision-making. The findings lay the groundwork for future research integrating this optimized hybrid feature subset into DL architectures, such as Vision Transformers, to further enhance model generalization, computational efficiency, and clinical applicability in precision oncology. Building upon these results, upcoming work should focus on expanding the dataset with longitudinal and multi-view mammographic data to capture temporal–spatial tumor evolution, incorporating explainable AI (XAI) tools to improve transparency and clinical trust, and developing physics-informed hybrid networks that combine mechanistic modeling with statistical and DL paradigms. Such integration could bridge the gap between quantitative radiomics and real-world diagnostic precision, contributing to more reliable breast cancer screening systems.

## Figures and Tables

**Table 1:** The linear feature’s evaluation results in the breast cancer detection using different statistical selection methods.

Linear Features	Statistical Test	Performance (%)	Accuracy ± SD (%)	Sensitivity ± SD (%)	Specificity ± SD (%)	F1-Score ± SD (%)
Mean intensity	*t*-test	87.0	88.0 ± 1.45	84.0 ± 0.59	89.0 ± 0.58	87.0 ± 0.60
	ANOVA	87.3	88.3 ± 1.4	84.2 ± 0.60	89.2 ± 0.60	87.3 ± 0.60
	MI	88.0	89.1 ± 1.3	85.0 ± 0.57	90.0 ± 0.58	88.0 ± 0.59
	EGM	91.0	92.0 ± 1	88.0 ± 0.48	93.0 ± 0.48	91.0 ± 0.48
	*t*-test + ANOVA	89.7	90.8 ± 1.2	86.5 ± 0.54	91.9 ± 0.55	89.6 ± 0.58
	*t*-test + MI	90.2	91.2 ± 1.1	87.0 ± 0.53	92.3 ± 0.53	90.1 ± 0.55
	*t*-test + EGM	91.5	92.5 ± 0.9	88.5 ± 0.41	93.5 ± 0.45	91.5 ± 0.45
	ANOVA + MI	90.4	91.4 ± 1	87.3 ± 0.50	92.5 ± 0.50	90.3 ± 0.50
	ANOVA + EGM	91.7	92.7 ± 0.9	88.7 ± 0.41	93.7 ± 0.42	91.7 ± 0.43
	MI + EGM	92.0	93.0 ± 0.88	89.0 ± 0.39	94.0 ± 0.40	92.0 ± 0.40
	**EGM + ANOVA +** ***t*** **-test + MI**	**92.3**	**93.3 **±** 0.84**	**89.3 **±** 0.20**	**94.3 **±** 0.3**	**92.3 **±** 0.38**
Variance	*t*-test	88.1	89.0 ± 1.35	85.3 ± 0.60	90.1 ± 0.62	88.0 ± 0.57
	ANOVA	88.4	89.3 ± 1.32	85.6 ± 0.57	90.4 ± 0.60	88.3 ± 0.56
	MI	89.2	90.2 ± 1.22	86.3 ± 0.57	91.1 ± 0.58	89.0 ± 0.54
	EGM	92.0	93.0 ± 0.9	89.0 ± 0.45	94.0 ± 0.48	92.0 ± 0.47
	*t*-test + ANOVA	90.8	91.6 ± 1	88.0 ± 0.55	92.8 ± 0.55	90.6 ± 0.52
	*t*-test + MI	91.1	92.0 ± 1	88.4 ± 0.49	93.1 ± 0.53	91.0 ± 0.50
	*t*-test + EGM	92.5	93.5 ± 0.82	89.5 ± 0.46	94.5 ± 0.50	92.5 ± 0.45
	ANOVA + MI	91.3	92.2 ± 0.98	88.6 ± 0.45	93.3 ± 0.45	91.2 ± 0.48
	ANOVA +EGM	92.6	93.6 ± 0.80	89.6 ± 0.39	94.6 ± 0.43	92.6 ± 0.40
	MI +EGM	93.0	94.0 ± 0.75	90.0 ± 0.40	95.0 ± 0.40	93.0 ± 0.39
	**EGM + ANOVA +** ***t*** **-test + MI**	**93.3**	**94.3 **±** 0.70**	**90.3 **±** 0.21**	**95.3 **±** 0.3**	**93.3 **±** 0.30**
Skewness	*t*-test	89.3	90.0 ± 1.20	87.0 ± 0.45	91.0 ± 0.45	89.0 ± 0.49
	ANOVA	89.6	90.3 ± 0.74	87.3 ± 0.46	91.3 ± 0.46	89.3 ± 0.47
	MI	90.3	91.2 ± 1.15	88.0 ± 0.48	92.0 ± 0.48	90.0 ± 0.46
	EGM	93.2	94.0 ± 1.05	90.7 ± 0.39	95.0 ± 0.40	93.0 ± 0.40
	*t*-test + ANOVA	91.7	92.4 ± 0.90	89.3 ± 0.43	93.5 ± 0.46	91.6 ± 0.45
	*t*-test + MI	92.1	92.8 ± 0.85	89.7 ± 0.40	93.9 ± 0.44	92.0 ± 0.40
	ANOVA + MI	92.3	93.0 ± 0.55	89.9 ± 0.39	94.1 ± 0.40	92.1 ± 0.40
	EGM + *t*-test	93.7	94.5 ± 0.68	91.2 ± 0.40	95.5 ± 0.40	93.5 ± 0.40
	EGM + ANOVA	93.8	94.6 ± 0.80	91.3 ± 0.36	95.6 ± 0.40	93.6 ± 0.35
	EGM + MI	94.2	95.0 ± 0.75	91.6 ± 0.35	96.0 ± 0.40	94.0 ± 0.31
	**EGM + ANOVA +** ***t*** **-test + MI**	**94.5**	**95.3 **±** 0.70**	**91.9 **±** 0.30**	**96.3 **±** 0.30**	**94.3 **±** 0.30**
Kurtosis	*t*-test	89.6	90.5 ± 1.15	87.0 ± 0.47	91.7 ± 0.48	89.0 ± 0.50
	ANOVA	89.8	90.7 ± 1.12	87.2 ± 0.43	91.9 ± 0.50	89.2 ± 0.47
	MI	90.6	91.6 ± 1.02	88.1 ± 0.44	92.8 ± 0.48	90.0 ± 0.45
	EGM	93.5	94.5 ± 0.72	91.0 ± 0.39	95.5 ± 0.38	93.0 ± 0.35
	*t*-test + ANOVA	92.4	93.1 ± 0.88	90.0 ± 0.46	94.2 ± 0.45	92.3 ± 0.42
	*t*-test + MI	92.8	93.5 ± 0.82	90.4 ± 0.40	94.6 ± 0.43	92.6 ± 0.40
	ANOVA + MI	93.0	93.7 ± 0.80	90.6 ± 0.39	94.8 ± 0.40	92.8 ± 0.37
	EGM + *t*-test	93.9	94.9 ± 0.66	91.4 ± 0.31	95.9 ± 0.36	93.4 ± 0.34
	EGM + ANOVA	94.0	95.0 ± 0.64	91.5 ± 0.30	96.0 ± 0.33	93.5 ± 0.32
	EGM + MI	94.3	95.3 ± 0.58	91.8 ± 0.30	96.3 ± 0.30	93.8 ± 0.30
	**EGM + ANOVA +** ***t*** **-test + MI**	**94.6**	**95.6 **±** 0.54**	**92.1 **±** 0.20**	**96.6 **±** 0.20**	**94.6 **±** 0.20**
Volume/ area	*t*-test	88.7	89.5 ± 1.30	86.0 ± 0.48	90.7 ± 0.49	88.5 ± 0.50
	ANOVA	89.0	89.8 ± 1.25	86.3 ± 0.46	91.0 ± 0.50	88.8 ± 0.46
	MI	89.8	90.7 ± 1.15	87.0 ± 0.43	91.8 ± 0.48	89.5 ± 0.45
	EGM	93.0	94.0 ± 0.78	90.0 ± 0.35	95.0 ± 0.39	93.0 ± 0.39
	*t*-test + ANOVA	91.3	92.1 ± 0.95	88.6 ± 0.40	93.3 ± 0.46	91.3 ± 0.43
	*t*-test + MI	91.8	92.6 ± 0.88	89.1 ± 0.40	93.8 ± 0.44	91.8 ± 0.40
	ANOVA + MI	92.0	92.8 ± 0.80	89.3 ± 0.39	94.0 ± 0.40	92.0 ± 0.38
	EGM + *t*-test	93.5	94.5 ± 0.70	90.5 ± 0.36	95.5 ± 0.37	93.5 ± 0.37
	EGM + ANOVA	93.7	94.7 ± 0.68	90.7 ± 0.35	95.7 ± 0.43	93.7 ± 0.35
	EGM + MI	94.0	95.0 ± 0.60	91.0 ± 0.31	96.0 ± 0.30	94.0 ± 0.30
	**EGM + ANOVA +** ***t*** **-test + MI**	**94.3**	**95.3 **±** 0.58**	**91.3 **±** 0.20**	**96.3 **±** 0.20**	**94.3 **±** 0.20**
Perimeter irregularity	*t*-test	89.4	90.2 ± 1.20	87.0 ± 0.45	91.2 ± 0.50	89.0 ± 0.48
	ANOVA	89.7	90.5 ± 1.15	87.3 ± 0.40	91.5 ± 0.48	89.3 ± 0.42
	MI	90.5	91.4 ± 1	88.2 ± 0.41	92.4 ± 0.46	90.0 ± 0.41
	EGM	93.5	94.3 ± 0.72	91.2 ± 0.31	95.3 ± 0.37	93.0 ± 0.30
	*t*-test + ANOVA	92.3	93.0 ± 0.88	90.3 ± 0.40	94.0 ± 0.44	92.0 ± 0.40
	*t*-test + MI	93.1	93.7 ± 0.80	91.2 ± 0.38	94.7 ± 0.42	92.7 ± 0.37
	ANOVA + MI	93.3	93.9 ± 0.78	91.4 ± 0.39	94.9 ± 0.40	92.9 ± 0.35
	EGM + *t*-test	93.9	94.8 ± 0.30	91.7 ± 0.30	95.8 ± 0.35	93.5 ± 0.27
	EGM + ANOVA	94.2	95.0 ± 0.62	91.9 ± 0.64	96.0 ± 0.43	93.7 ± 0.25
	EGM + MI	94.5	95.3 ± 0.62	92.2 ± 0.25	96.3 ± 0.30	94.0 ± 0.25
	**EGM + ANOVA +** ***t*** **-test + MI**	94.8	**95.6 **±** 0.52**	**92.5 **±** 0.20**	**96.6 **±** 0.20**	**94.5 **±** 0.20**
Haralick entropy	EGM	94.3	95.0 ± 0.48	92.0 ± 0.31	96.0 ± 0.30	94.0 ± 0.43
	*t*-test	90.3	91.0 ± 1.05	88.0 ± 0.31	92.0 ± 0.40	90.0 ± 0.34
	ANOVA	90.6	91.3 ± 1	88.3 ± 0.40	92.3 ± 0.40	90.3 ± 0.40
	MI	91.3	92.0 ± 0.95	89.0 ± 0.39	93.0 ± 0.40	91.0 ± 0.41
	*t*-test + ANOVA	93.4	94.0 ± 0.72	91.5 ± 0.39	95.0 ± 0.36	93.0 ± 0.40
	*t*-test + MI	93.7	94.3 ± 0.68	91.8 ± 0.39	95.3 ± 0.34	93.3 ± 0.37
	ANOVA + MI	93.9	94.5 ± 0.65	92.0 ± 0.34	95.5 ± 0.32	93.5 ± 0.35
	EGM + *t*-test	94.7	95.5 ± 0.58	92.5 ± 0.30	96.5 ± 0.30	94.5 ± 0.27
	EGM + ANOVA	94.8	95.6 ± 0.55	92.6 ± 0.29	96.6 ± 0.30	94.6 ± 0.25
	EGM + MI	95.3	96.0 ± 0.52	93.0 ± 0.25	97.0 ± 0.30	95.0 ± 0.25
	**EGM + ANOVA +** ***t*** **-test + MI**	**95.6**	**96.3 **±** 0.48**	**93.3 **±** 0.20**	**97.3 **±** 0.20**	**95.3 **±** 0.20**
Intensity distribution	*t*-test	88.1	89.0 ± 0.35	85.5 ± 0.25	90.0 ± 0.56	88.0 ± 0.21
	ANOVA	88.4	89.3 ± 1.30	85.8 ± 0.34	90.3 ± 0.50	88.3 ± 0.36
	MI	89.2	90.2 ± 1.22	86.6 ± 0.40	91.2 ± 0.40	89.0 ± 0.40
	EGM	92.0	93.0 ± 0.92	89.0 ± 0.31	94.0 ± 0.30	92.0 ± 0.30
	*t*-test + MI	91.0	91.9 ± 0.98	88.3 ± 0.34	93.0 ± 0.40	90.8 ± 0.34
	ANOVA + MI	91.2	92.1 ± 0.95	88.5 ± 0.34	93.2 ± 0.40	91.0 ± 0.30
	EGM + *t*-test	92.5	93.5 ± 0.80	89.5 ± 0.25	94.5 ± 0.28	92.5 ± 0.28
	EGM + ANOVA	92.7	93.7 ± 0.78	89.7 ± 0.25	94.7 ± 0.25	92.7 ± 0.25
	EGM + MI	93.0	94.0 ± 0.74	90.0 ± 0.25	95.0 ± 0.23	93.0 ± 0.21
	**EGM + ANOVA +** ***t*** **-test + MI**	**93.3**	**94.3 **±** 0.70**	**90.3 **±** 0.20**	**95.3 **±** 0.20**	**93.3 **±** 0.20**
Temporal variation	*t*-test	89.3	90.0 ± 1.18	87.0 ± 0.25	91.0 ± 0.40	89.0 ± 0.21
	EGM	93.1	94.0 ± 0.76	90.5 ± 0.24	95.0 ± 0.30	93.0 ± 0.25
	ANOVA	89.6	90.3 ± 1.12	87.3 ± 0.23	91.3 ± 0.40	89.3 ± 0.23
	MI	90.3	91.1 ± 1	88.1 ± 0.26	92.1 ± 0.40	90.0 ± 0.28
	*t*-test + ANOVA	91.9	92.8 ± 0.90	89.4 ± 0.26	93.9 ± 0.30	91.7 ± 0.28
	*t*-test + MI	92.4	93.2 ± 0.84	89.8 ± 0.25	94.3 ± 0.30	92.1 ± 0.24
	ANOVA + MI	92.6	93.4 ± 0.80	90.0 ± 0.21	94.5 ± 0.30	92.3 ± 0.20
	EGM + *t*-test	93.6	94.5 ± 0.68	91.0 ± 0.21	95.5 ± 0.28	93.5 ± 0.22
	EGM + ANOVA	93.8	94.7 ± 0.64	91.2 ± 0.21	95.7 ± 0.27	93.7 ± 0.21
	EGM + MI	94.1	95.0 ± 0.60	91.5 ± 0.25	96.0 ± 0.25	94.0 ± 0.20
	**EGM + ANOVA +** ***t*** **-test + MI**	**94.3**	**95.3 **±** 0.56**	**91.8 **±** 0.20**	**96.3 **±** 0.20**	**94.3 **±** 0.20**

**Notes:** Bold: Indicates the highest values and the framework yielding the highest values. Red: Indicates the linear features leading to the highest values.

**Table 2:** The nonlinear feature evaluation results in breast cancer detection using different statistical selection methods.

NonLinear Features	Statistical Test	Performance (%)	Accuracy ± SD (%)	Sensitivity ± SD (%)	Specificity ± SD (%)	F1-Score ± SD (%)
Shannon entropy	*t*-test	93.5	94.6 ± 0.50	95.2 ± 0.30	94.0 ± 0.50	94.6 ± 0.50
	ANOVA	93.6	94.7 ± 0.48	95.3 ± 0.30	94.1 ± 0.70	94.7 ± 0.60
	MI	93.6	94.7 ± 0.40	95.3 ± 0.40	94.1 ± 0.38	94.7 ± 0.38
	EGM	93.7	94.8 ± 0.40	95.4 ± 0.44	94.2 ± 0.44	94.8 ± 0.44
	*t*-test + ANOVA	93.6	94.7 ± 0.43	95.3 ± 0.30	94.1 ± 0.42	94.7 ± 0.40
	*t*-test + MI	93.7	94.8 ± 0.40	95.4 ± 0.30	94.2 ± 0.39	94.8 ± 0.39
	*t*-test + EGM	93.8	94.9 ± 0.44	95.5 ± 0.20	94.3 ± 0.40	94.9 ± 0.44
	ANOVA + MI	93.7	94.8 ± 0.40	95.4 ± 0.30	94.2 ± 0.39	94.8 ± 0.39
	ANOVA + EGM	93.8	94.9 ± 0.44	95.5 ± 0.26	94.3 ± 0.45	94.9 ± 0.46
	MI + EGM	93.9	95.0 ± 0.40	95.6 ± 0.20	94.4 ± 0.44	95.0 ± 0.44
	**EGM + ANOVA +** ***t*** **-test + MI**	**94.0**	**95.1 **±** 0.30**	**95.7 **±** 0.20**	**94.5 **±** 0.30**	**95.1 **±** 0.30**
Tsallis entropy	*t*-test	92.8	93.9 ± 0.60	94.5 ± 0.40	93.2 ± 0.67	93.9 ± 0.65
	ANOVA	92.9	94.0 ± 0.58	94.6 ± 0.40	93.3 ± 0.56	94.0 ± 0.56
	MI	92.9	94.0 ± 0.56	94.6 ± 0.40	93.3 ± 0.55	94.0 ± 0.45
	EGM	93.0	94.1 ± 0.46	94.7 ± 0.40	93.4 ± 0.47	94.1 ± 0.48
	*t*-test + ANOVA	92.9	94.0 ± 0.50	94.6 ± 0.51	93.3 ± 0.52	94.0 ± 0.52
	*t*-test + MI	93.0	94.1 ± 0.50	94.7 ± 0.44	93.4 ± 0.42	94.1 ± 0.45
	*t*-test + EGM	93.1	94.2 ± 0.44	94.8 ± 0.40	93.5 ± 0.45	94.2 ± 0.45
	ANOVA + MI	93.0	94.1 ± 0.46	94.7 ± 0.43	93.4 ± 0.42	94.1 ± 0.40
	ANOVA + EGM	93.1	94.2 ± 0.40	94.8 ± 0.30	93.5 ± 0.40	94.2 ± 0.41
	MI + EGM	93.2	94.3 ± 0.35	94.9 ± 0.31	93.6 ± 0.33	94.3 ± 0.32
	**EGM + ANOVA +** ***t*** **-test + MI**	**93.3**	**94.4 **±** 0.30**	**95.0 **±** 0.20**	**93.7 **±** 0.30**	**94.4 **±** 0.30**
Renyi entropy	*t*-test	93.0	94.1 ± 0.58	94.7 ± 0.57	93.4 ± 0.56	94.1 ± 0.58
	ANOVA	93.1	94.2 ± 0.56	94.8 ± 0.52	93.5 ± 0.54	94.2 ± 0.55
	MI	93.1	94.2 ± 0.54	94.8 ± 0.51	93.5 ± 0.53	94.2 ± 0.52
	EGM	93.2	94.3 ± 0.40	94.9 ± 0.42	93.6 ± 0.43	94.3 ± 0.40
	*t*-test + ANOVA	93.1	94.2 ± 0.50	94.8 ± 0.51	93.5 ± 0.53	94.2 ± 0.51
	*t*-test + MI	93.2	94.3 ± 0.38	94.9 ± 0.35	93.6 ± 0.36	94.3 ± 0.37
	*t*-test + EGM	93.3	94.4 ± 0.35	95.0 ± 0.31	93.7 ± 0.36	94.4 ± 0.33
	ANOVA + MI	93.2	94.3 ± 0.30	94.9 ± 0.31	93.6 ± 0.35	94.3 ± 0.36
	ANOVA + EGM	93.3	94.4 ± 0.35	95.0 ± 0.36	93.7 ± 0.36	94.4 ± 0.33
	MI + EGM	93.4	94.5 ± 0.30	95.1 ± 0.31	93.8 ± 0.31	94.5 ± 0.31
	**EGM + ANOVA +** ***t*** **-test + MI**	**93.5**	**94.6 **±** 0.30**	**95.2 **±** 0.30**	**93.9 **±** 0.30**	**94.6 **±** 0.30**
Fractal Dimension (FD)	*t*-test	92.5	93.6 ± 0.70	94.6 ± 0.75	92.6 ± 0.75	93.6 ± 0.70
	ANOVA	92.6	93.7 ± 0.68	94.7 ± 0.69	92.7 ± 0.67	93.7 ± 0.65
	MI	92.7	93.8 ± 0.65	94.8 ± 0.65	92.8 ± 0.46	93.8 ± 0.61
	EGM	92.9	94.0 ± 0.60	95.0 ± 0.58	93.0 ± 0.53	94.0 ± 0.50
	*t*-test + ANOVA	92.8	93.9 ± 0.58	94.9 ± 0.56	92.9 ± 0.57	93.9 ± 0.55
	*t*-test + MI	92.9	94.0 ± 0.50	95.0 ± 0.48	93.0 ± 0.46	94.0 ± 0.45
	*t*-test + EGM	93.1	94.2 ± 0.45	95.2 ± 0.43	93.1 ± 0.44	94.2 ± 0.47
	ANOVA + MI	93.0	94.1 ± 0.35	95.1 ± 0.37	93.0 ± 0.34	94.1 ± 0.39
	ANOVA + EGM	93.2	94.3 ± 0.48	95.3 ± 0.44	93.2 ± 0.41	94.3 ± 0.49
	MI + EGM	93.3	94.4 ± 0.35	95.4 ± 0.36	93.3 ± 0.34	94.4 ± 0.40
	**EGM + ANOVA +** ***t*** **-test + MI**	**93.4**	**94.5 **±** 0.30**	**95.5 **±** 0.29**	**93.4 **±** 0.30**	**94.5 **±** 0.30**
Wavelet entropy	*t*-test	96.2	97.4 ± 0.45	98.4 ± 0.44	96.2 ± 0.46	97.4 ± 0.48
	ANOVA	96.3	97.5 ± 0.40	98.5 ± 0.39	96.3 ± 0.37	97.5 ± 0.40
	MI	96.3	97.5 ± 0.40	98.5 ± 0.41	96.3 ± 0.43	97.5 ± 0.40
	EGM	96.4	97.6 ± 0.36	98.6 ± 0.34	96.4 ± 0.33	97.6 ± 0.34
	*t*-test + ANOVA	96.3	97.5 ± 0.36	98.5 ± 0.34	96.3 ± 0.30	97.5 ± 0.34
	*t*-test + MI	96.4	97.6 ± 0.35	98.6 ± 0.29	96.4 ± 0.27	97.6 ± 0.30
	*t*-test + EGM	96.5	97.7 ± 0.28	98.7 ± 0.25	96.5 ± 0.24	97.7 ± 0.27
	ANOVA + MI	96.4	97.6 ± 0.22	98.6 ± 0.21	96.4 ± 0.23	97.6 ± 0.24
	ANOVA + EGM	96.5	97.7 ± 0.20	98.7 ± 0.21	96.5 ± 0.22	97.7 ± 0.20
	MI + EGM	96.6	97.8 ± 0.22	98.8 ± 0.21	96.6 ± 0.21	97.8 ± 0.23
	**EGM + ANOVA +** ***t*** **-test + MI**	**96.7**	**97.9 **±** 0.20**	**98.9 **±** 0.20**	**96.7 **±** 0.21**	**97.9 **±** 0.21**
Local phase congruency	*t*-test	96.4	97.6 ± 0.40	98.6 ± 0.40	96.4 ± 0.43	97.6 ± 0.44
	ANOVA	96.4	97.6 ± 0.30	98.6 ± 0.31	96.4 ± 0.30	97.6 ± 0.36
	MI	96.5	97.7 ± 0.26	98.7 ± 0.26	96.5 ± 0.27	97.7 ± 0.25
	EGM	96.6	97.8 ± 0.24	98.8 ± 0.23	96.6 ± 0.21	97.8 ± 0.24
	*t*-test + ANOVA	96.5	97.7 ± 0.23	98.7 ± 0.23	96.5 ± 0.21	97.7 ± 0.20
	*t*-test + MI	96.6	97.8 ± 0.20	98.8 ± 0.12	96.6 ± 0.14	97.8 ± 0.15
	*t*-test + EGM	96.7	97.9 ± 0.15	98.9 ± 0.13	96.7 ± 0.14	97.9 ± 0.15
	ANOVA + MI	96.6	97.8 ± 0.20	98.8 ± 0.20	96.6 ± 0.23	97.8 ± 0.20
	ANOVA + EGM	96.7	97.9 ± 0.11	98.9 ± 0.12	96.7 ± 0.16	97.9 ± 0.15
	MI + EGM	96.8	98.0 ± 0.10	99.0 ± 0.11	96.8 ± 0.12	98.0 ± 0.10
	**EGM + ANOVA +** ***t*** **-test + MI**	**96.9**	**98.1 **±** 0.10**	**99.1 **±** 0.10**	**96.9 **±** 0.10**	**98.1 **±** 0.10**
Nonlinear diffusion	*t*-test	95.6	96.8 ± 0.40	97.8 ± 0.41	95.6 ± 0.40	96.8 ± 0.40
	ANOVA	95.7	96.9 ± 0.35	97.9 ± 0.35	95.7 ± 0.34	96.9 ± 0.33
	MI	95.8	97.0 ± 0.30	98.0 ± 0.35	95.8 ± 0.31	97.0 ± 0.30
	EGM	95.9	97.1 ± 0.27	98.1 ± 0.26	95.9 ± 0.23	97.1 ± 0.30
	*t*-test + ANOVA	95.8	97.0 ± 0.25	98.0 ± 0.22	95.8 ± 0.23	97.0 ± 0.26
	*t*-test + MI	95.9	97.1 ± 0.30	98.1 ± 0.31	95.9 ± 0.35	97.1 ± 0.20
	*t*-test + EGM	96.0	97.2 ± 0.27	98.2 ± 0.25	96.0 ± 0.21	97.2 ± 0.20
	ANOVA + MI	95.9	97.1 ± 0.25	98.1 ± 0.23	95.9 ± 0.22	97.1 ± 0.26
	ANOVA + EGM	96.0	97.2 ± 0.23	98.2 ± 0.21	96.0 ± 0.24	97.2 ± 0.23
	MI + EGM	96.1	97.3 ± 0.21	98.3 ± 0.19	96.1 ± 0.20	97.3 ± 0.20
	**EGM + ANOVA +** ***t*** **-test + MI**	**96.2**	**97.4 **±** 0.20**	**98.4 **±** 0.20**	**96.2 **±** 0.20**	**97.4 **±** 0.20**
Clustering coefficient	*t*-test	95.2	96.4 ± 0.40	97.4 ± 0.40	95.2 ± 0.41	96.4 ± 0.42
	ANOVA	95.3	96.5 ± 0.33	97.5 ± 0.35	95.3 ± 0.36	96.5 ± 0.37
	MI	95.4	96.6 ± 0.31	97.6 ± 0.30	95.4 ± 0.32	96.6 ± 0.30
	EGM	95.5	96.7 ± 0.30	97.7 ± 0.29	95.5 ± 0.27	96.7 ± 0.30
	*t*-test + ANOVA	95.4	96.6 ± 0.30	97.6 ± 0.29	95.4 ± 0.30	96.6 ± 0.30
	*t*-test + MI	95.5	96.7 ± 0.28	97.7 ± 0.25	95.5 ± 0.26	96.7 ± 0.27
	*t*-test + EGM	95.6	96.8 ± 0.25	97.8 ± 0.25	95.6 ± 0.27	96.8 ± 0.26
	ANOVA + MI	95.5	96.7 ± 0.21	97.7 ± 0.21	95.5 ± 0.24	96.7 ± 0.20
	ANOVA + EGM	95.6	96.8 ± 0.20	97.8 ± 0.20	95.6 ± 0.20	96.8 ± 0.21
	MI + EGM	95.7	96.9 ± 0.20	97.9 ± 0.20	95.7 ± 0.19	96.9 ± 0.17
	**EGM + ANOVA +** ***t*** **-test + MI**	**95.8**	**97.0 **±** 0.20**	**98.0 **±** 0.20**	**95.8 **±** 0.20**	**97.0 **±** 0.20**
Laplacian spectrum features	*t*-test	96.1	97.3 ± 0.30	98.3 ± 0.30	96.1 ± 0.31	97.3 ± 0.33
	ANOVA	96.2	97.4 ± 0.28	98.4 ± 0.27	96.2 ± 0.24	97.4 ± 0.26
	MI	96.2	97.4 ± 0.25	98.4 ± 0.24	96.2 ± 0.23	97.4 ± 0.25
	EGM	96.3	97.5 ± 0.21	98.5 ± 0.21	96.3 ± 0.20	97.5 ± 0.19
	*t*-test + ANOVA	96.2	97.4 ± 0.30	98.4 ± 0.48	96.2 ± 0.21	97.4 ± 0.21
	*t*-test + MI	96.3	97.5 ± 0.20	98.5 ± 0.21	96.3 ± 0.21	97.5 ± 0.23
	*t*-test + EGM	96.4	97.6 ± 0.20	98.6 ± 0.20	96.4 ± 0.20	97.6 ± 0.20
	ANOVA + MI	96.3	97.5 ± 0.19	98.5 ± 0.17	96.3 ± 0.13	97.5 ± 0.11
	ANOVA + EGM	96.4	97.6 ± 0.20	98.6 ± 0.19	96.4 ± 0.17	97.6 ± 0.15
	MI + EGM	96.5	97.7 ± 0.20	98.7 ± 0.21	96.5 ± 0.17	97.7 ± 0.16
	**EGM + ANOVA +** ***t*** **-test + MI**	**96.6**	**97.8 **±** 0.20**	**98.8 **±** 0.17**	**96.6 **±** 0.15**	**97.8 **±** 0.13**
Local Binary Patterns	*t*-test	94.5	95.6 ± 0.55	96.2 ± 0.60	95.0 ± 0.55	95.6 ± 0.53
	ANOVA	94.6	95.7 ± 0.45	96.3 ± 0.40	95.1 ± 0.43	95.7 ± 0.44
	MI	94.7	95.8 ± 0.39	96.4 ± 0.36	95.2 ± 0.33	95.8 ± 0.34
	EGM	94.8	95.9 ± 0.40	96.5 ± 0.35	95.3 ± 0.33	95.9 ± 0.30
	*t*-test + ANOVA	94.7	95.8 ± 0.41	96.4 ± 0.36	95.2 ± 0.34	95.8 ± 0.32
	*t*-test + MI	94.8	95.9 ± 0.36	96.5 ± 0.38	95.3 ± 0.34	95.9 ± 0.35
	*t*-test + EGM	94.9	96.0 ± 0.32	96.6 ± 0.31	95.4 ± 0.30	96.0 ± 0.32
	ANOVA + MI	94.8	95.9 ± 0.40	96.5 ± 0.35	95.3 ± 0.36	95.9 ± 0.33
	ANOVA + EGM	94.9	96.0 ± 0.30	96.6 ± 0.31	95.4 ± 0.33	96.0 ± 0.30
	MI + EGM	95.0	96.1 ± 0.20	96.7 ± 0.30	95.5 ± 0.27	96.1 ± 0.37
	**EGM + ANOVA +** ***t*** **-test + MI**	**95.1**	**96.2 **±** 0.37**	**96.8 **±** 0.29**	**95.6 **±** 0.30**	**96.2 **±** 0.35**

**Notes:** Bold: Indicates the highest values and the framework yielding the highest values. Blue: Indicates the nonlinear features leading to the highest values.

**Table 3:** A summary of the evaluation results of explored features: average (Δ) accuracy and (Δ) performance among statistical selection methods and combinations.

Feature	Type	ΔAccuracy (%)	ΔPerformance (%)
**Haralick entropy**	Linear	**94.14**	**93.45**
**Kurtosis**	Linear	**93.49**	**92.59**
**Perimeter irregularity**	Linear	**93.43**	**92.65**
**Temporal Variation**	Linear	**93.12**	**92.27**
**Skewness**	Linear	**93.01**	**92.25**
**Volume/area**	Linear	**92.82**	**91.92**
Variance	Linear	92.06	91.12
Intensity distribution	Linear	92.05	91.09
Mean intensity	Linear	91.12	90.10
**Local phase congruency**	Nonlinear	**97.81**	**96.61**
**Wavelet entropy**	Nonlinear	**97.62**	**96.42**
**Laplacian spectrum features**	Nonlinear	**97.52**	**96.32**
**Nonlinear diffusion**	Nonlinear	**97.10**	**95.90**
**Clustering coefficient**	Nonlinear	**96.70**	**95.50**
**Tsallis entropy**	Nonlinear	**96.55**	**95.45**
**Shannon’s entropy**	Nonlinear	**96.53**	**95.10**
**Rényi entropy**	Nonlinear	**96.30**	**95.20**
Local Binary Patterns	Nonlinear	95.90	94.80
Fractal Dimension (FD)	Nonlinear	94.05	92.95

**Notes:** Bold: Indicates highest values and the features yielding the highest values.

## Data Availability

Data are contained within the article.
